# Serum peptidome profiling for the diagnosis of colorectal cancer: discovery and validation in two independent cohorts

**DOI:** 10.18632/oncotarget.19587

**Published:** 2017-07-26

**Authors:** Hao Wang, Chenghua Luo, Shengtao Zhu, Honghong Fang, Qing Gao, Siqi Ge, Haixia Qu, Qingwei Ma, Hongwei Ren, Youxin Wang, Wei Wang

**Affiliations:** ^1^ Beijing Key Laboratory of Clinical Epidemiology, School of Public Health, Capital Medical University, Beijing 100069, China; ^2^ Department of Retroperitoneal Tumors Surgery, Peking University International Hospital, Beijing 102206, China; ^3^ Department of Gastroenterology, Beijing Friendship Hospital, Capital Medical University, Beijing 100069, China; ^4^ National Center for Clinical Medical Research of Digestive Diseases, Beijing 100069, China; ^5^ School of Medical and Health Sciences, Edith Cowan University, Perth 6027, Australia; ^6^ Bioyong (Beijing) Technology Co., Ltd., Beijing 100085, China; ^7^ School of Life Sciences, Peking University, Beijing 100871, China

**Keywords:** colorectal cancer, peptidome, MALDI-TOF MS, diagnosis panel

## Abstract

Colorectal cancer (CRC) is one of the most common malignant neoplasms worldwide. Except for the existing fecal occult blood test, colonoscopy and sigmoidoscopy, no widely accepted *in vitro* diagnostic methods have been available. To identify potential peptide biomarkers for CRC, serum samples from a discovery cohort (100 CRC patients and 100 healthy controls) and an independent validation cohort (91 CRC patients and 91 healthy controls) were collected. Peptides were fractionated by weak cation exchange magnetic beads (MB-WCX) and analysed by matrix-assisted laser desorption/ionization time-of-flight mass spectrometry (MALDI-TOF MS). Five peptides (peaks at *m/z* 1895.3, 2020.9, 2080.7, 2656.8 and 3238.5) were identified as candidate biomarkers for CRC. A diagnostic panel based on the five peptides can discriminate CRC patients from healthy controls, with an accuracy of 91.8%, sensitivity of 95.6%, and specificity of 87.9% in the validation cohort. Peptide peaks at *m/z* 1895.3, 2020.9 and 3238.5 were identified as the partial sequences of complement component 4 (C4), complement component 3 (C3) and fibrinogen α chain (FGA), respectively. This study potentiated peptidomic analysis as a promising *in vitro* diagnostic tool for diagnosis of CRC. The identified peptides suggest the involvement of the C3, C4 and FGA in CRC pathogenesis.

## INTRODUCTION

Colorectal cancer (CRC) is the 3^rd^ most common cancer among men (after lung and prostate cancer) and the 2^nd^ most frequent cancer among women after breast cancer [1]. In 2012, 1.4 million new cases of CRC and nearly 0.7 million CRC-related deaths occurred worldwide [2]. The 5-years relative survival rate for localized CRC is 90.3%, and it decreases to 70.4% and 12.5% when the cancer has spread to the adjacent organs and distant organs, respectively [3]. Early diagnosis of CRC is an effective way to prolong the lives of CRC patients [3].

The American Cancer Society (ACS) recommends that individuals who are over 50 years old should schedule one of the following screenings: (1) a high-sensitivity fecal occult blood test (FOBT) every year, (2) a stool DNA test every 3 years, (3) a flexible sigmoidoscopy (FSIG) every 5 years, (4) a double-contrast barium enema every 5 years, (5) a computed tomography (CT) colonography every 5 years, or (6) a colonoscopy every 10 years [4]. However, due to the discomfort or high cost of these screening methods, only 55% of subjects aged 50 to 64 years have undergone a CRC screening test as suggested [5]. An examination of the entire colon by colonoscopy remains the golden standard for CRC screening, but people are hesitant to schedule a colonoscopy examination due to the complicated bowel preparation, associated discomfort, potential complications and high cost [6–9]. Although flexible sigmoidoscopy is less invasive than colonoscopy, this method is unable to examine the entire colon [10]. FOBT is non-invasive and economical, but it has relatively low sensitivity [11]. Carcinoembryonic antigen (CEA) has been extensively used as a blood-based marker for CRC prognosis [12], but it cannot be used as a diagnostic marker due to its relatively low specificity [13]. Currently, new blood-based tests that are accurate, safe, inexpensive, widely available, and associated with minimum patient discomfort are urgently required for the diagnosis of CRC.

The low-molecular-weight (LMW; ≤10kDa) serum peptidome represents the array of endogenous peptides that present in both intracellular and extracellular space of the body [14]. It contains several physiologically important peptides, such as peptide hormones, peptide metabolic products and proteolytic fragments of larger precursor proteins [15]. The proteolytic degradative patterns in the serum peptidome, often refer to as peptidome signature or fingerprint, hold important information about many physiological and pathological processes, such as aging [16], type 2 diabetes [17] and Alzheimer's disease [18]. The progress of tumor's malignancy is accompanied by alterations in exoproteases activities, affecting the constitution of endogenous peptides that can indicate the presence/absence of cancer [19]. The profiling of the serum peptidome has been used for the diagnosis of CRC in several studies [20–30]. Fan *et al* (2006) recruited 72 CRC patients and 65 healthy controls and randomly divided them into two groups: a model construction group and a validation group [20]. They established a diagnostic model with two peptides that yielded a sensitivity of 94.74% and a specificity of 100% in the model construction phase and a sensitivity of 94.12% and a specificity of 100% in the validation phase [20]. Several peptidome diagnostic models for CRC were also created by Deng *et al* (2013) [21], Liu *et al* (2006) [24] and Pietrowska *et al* (2012) [25]. However, these studies were hampered with small sample sizes, lacked independent validation or peptide identification.

The cancer-related biomarkers occur in blood at very low concentration levels. Immunoglobulins, albumin and other 20 proteins that make up approximately 99% of the protein content of serum can mask other proteins or peptides [15]. Therefore, it is imperative to eliminate all these abundant proteins before peptides profiling. Weak cation exchange magnetic beads (MB-WCX) method is one of the established fractionation methods that have high capturing ability of low abundance proteins or peptides in serum samples [31]. Proteomic/peptidomic studies necessitate a sensitive and high-throughput technique. Both matrix-assisted laser desorption/ionization time-of-flight mass spectrometry (MALDI-TOF MS) and liquid chromatography-tandem mass spectrometry (LC-MS/MS) have been applied widely to the analysis of serum/plasma, saliva and urine samples to diagnose human diseases as well as for the identification of potential biomarkers of health status [32, 33].

This study aimed to determine serum peptides biomarkers for CRC by MALDI-TOF MS combined with MB-WCX. A peptide diagnostic panel based on a set of potential peptide biomarkers was generated from a discovery cohort, and then further tested in another independent validation cohort. The identification of these peptides was performed using LC-MS/MS.

## RESULTS

### Characteristics of participants

The demographic and clinical characteristics of the participants in the two independent cohorts are summarized in Table 1. No significant differences were found in the distributions of age and gender between the CRC patients and controls (all *P* > 0.05). In the discovery cohort, 15.0% of the patients had early stage cancers (tumor-node-metastasis (TNM) stage I or II), where the remaining 85.0% had advanced stage cancers (TNM stage III or IV). In the validation cohort, 15.4% of the patients had early stage cancers (TNM stage I or II) and the remaining 84.6% had advanced stage cancers (TNM stage III or IV).

**Table 1:** Demographic and clinical characteristics of the participants

Characteristics	Discovery cohort			Validation cohort		
	CRC patients	Controls	*P*-value	CRC patients	Controls	*P*-value
Participants numbers	100	100	-	91	91	-
Age (years)	63.65±11.61	61.36±8.30	0.110	63.03±12.72	62.35±12.03	0.771
Gender (male/female)	57/43	57/43	1.000	50/41	50/41	1.000
TNM stage						
I (%)	3 (3.0%)	-	-	5 (5.5%)	-	-
II (%)	12 (12.0%)	-	-	9 (9.9%)	-	-
III (%)	58 (58.0%)	-	-	29 (31.9%)	-	-
IV (%)	27(27.0%)	-	-	48 (52.7%)	-	-

Student *t* test for age and chi-square test for gender. *P*-value lower than 0.05 was considered statistically significant.

### Selection of candidate peptides

**Figure 1:**
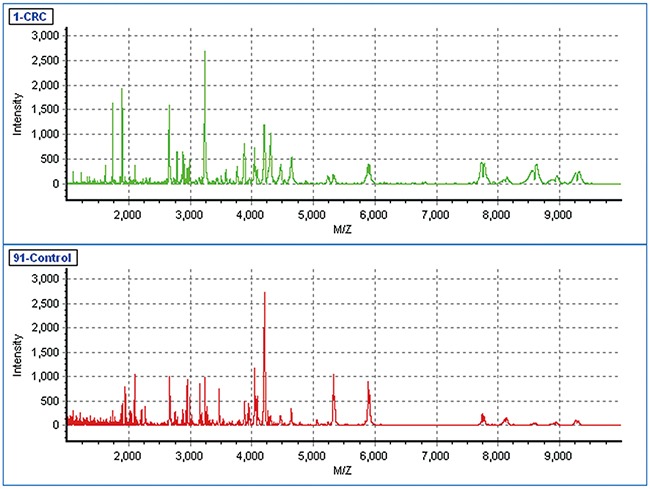
The MALDI-TOF spectra of the serum samples from a CRC patient and a healthy control The green line indicated the mass spectra of a CRC patient and the red line indicated the mass spectra of a healthy individual. X-axis, mass-to-charge ratio (*m/z*); Y-axis, relative intensity.

**Figure 2:**
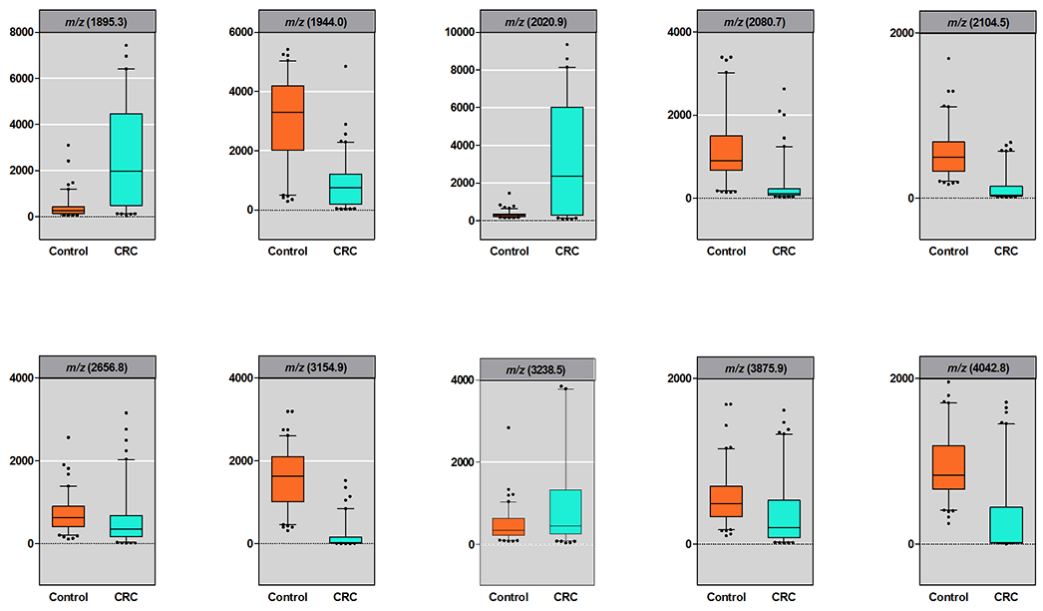
Box plots of peaks intensity in discovery cohort Results are presented as box and whisker plots: median, middle lines; 25-75th percentiles, rectangles; range, lines; outliers, markers.

The discovery cohort was used to select candidate peptides. The mass spectra of the serum samples from 100 CRC patients and 100 controls were obtained using MALDI-TOF MS (Figure 1). Among the 224 peptide peaks detected in the *m/z* range from 1,000 to 10,000, 22 peaks were detected in at least 50% of the serum samples. Out of these 22 peptide peaks, 10 peaks (*m/z* 1895.3, 1944.0, 2020.9, 2080.7, 2104.5, 2656.8, 3154.9, 3238.5, 3875.9 and 4042.8) in the patients were significantly different from those in the controls and were selected as candidate peptides for further analysis (*P* < 0.05). Among these 10 candidate peptides, 3 peptides (peaks at *m/z* 1895.3, 2020.9 and 3238.5) were at significantly higher levels, whereas the remaining 7 peptides (peaks at *m/z* 1944.0, 2080.7, 2104.5, 2656.8, 3154.9, 3875.9 and 4042.8) were at significantly lower levels in CRC patients compared with those of the controls (Figure 2). The area under the curve (AUC) was calculated to show the discriminatory power of these 10 candidate peptides, resulting a range from 0.623 to 0.980 of AUC in the discovery cohort (Table 2).

**Table 2:** Characteristics of candidate biomarkers in both discovery and validation cohorts

*m/z*	Tendency^1^	Discovery cohort					Validation cohort				
		Ave (C)^2^	Ave (N)^3^	*P*-value^4^	AUC^5^	95% CI^6^	Ave(C)^2^	Ave (N)^3^	*P*-value^4^	AUC^5^	95% CI^6^
1895.3	↑	2664.8	374.7	<0.001	0.838	(0.780-0.895)	2949.0	499.7	<0.001	0.845	(0.783-0.906)
1944.0	↓	858.5	3103.8	<0.001	0.906	(0.863-0.949)	918.3	2741.0	<0.001	0.841	(0.782-0.901)
2020.9	↑	3328.8	320.5	<0.001	0.746	(0.671-0.822)	4562.3	911.0	<0.001	0.823	(0.754-0.892)
2080.7	↓	295.8	1187.9	<0.001	0.896	(0.849-0.942)	221.2	1016.4	<0.001	0.893	(0.844-0.943)
2104.5	↓	132.8	544.7	<0.001	0.906	(0.864-0.948)	90.0	511.1	<0.001	0.934	(0.896-0.971)
2656.8	↓	565.6	704.3	<0.001	0.673	(0.597-0.750)	471.7	696.4	<0.001	0.699	(0.621-0.777)
3154.9	↓	145.8	1566.3	<0.001	0.980	(0.963-0.996)	132.6	1330.0	<0.001	0.918	(0.872-0.963)
3238.5	↑	1015.9	451.2	<0.001	0.623	(0.545-0.701)	680.4	456.9	0.456	0.535	(0.450-0.619)
3875.9	↓	349.7	557.3	<0.001	0.733	(0.660-0.806)	248.3	509.1	<0.001	0.759	(0.685-0.834)
4042.8	↓	271.5	940.5	<0.001	0.859	(0.802-0.916)	112.1	824.1	<0.001	0.897	(0.849-0.945)

^1^ Tendency↑represent the peaks intensity of CRC patients was higher than the controls,↓represent the peaks intensity of the CRC patients was lower than the controls; ^2^ Average intensity of peaks for the CRC patients; ^3^ Average intensity of peaks for the controls; ^4^ *P*-value calculated with the Wilcoxon test; ^5^ Area under ROC curve; ^6^ 95% confidence intervals; *P*-value lower than 0.05 was considered statistically significant.

### Establishment of the peptide diagnostic panel

**Figure 3:**
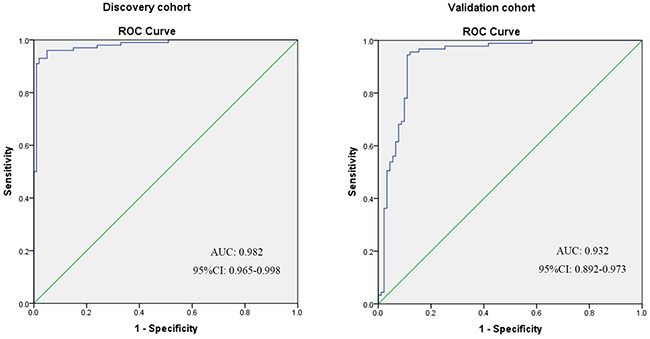
ROC curves of peptide diagnostic panel in the discovery and validation cohorts ROC curves illustrating the performance of peptide diagnostic panel in discriminating CRC patients from healthy controls.

To improve diagnostic accuracy for CRC, a multivariate binary logistic regression analysis was performed to establish a diagnostic panel with these 10 candidate peptides. The stepwise method (entry criteria: *P* < 0.05 and exclusion criteria: *P* > 0.10) was used to select the best panel of peptides. Consequently, 5 peptides (peaks at *m/z* 1895.3, 2020.9, 2080.7, 2656.8, and 3238.5) were selected in the diagnostic panel (Table 3). Receiver operating characteristic (ROC) curve and the AUC were used to assess the diagnostic performance of the panel. The panel yielded an accuracy of 95.5%, sensitivity of 96.0%, specificity of 95.0%, and AUC of 0.982, indicating a high discriminatory power (Figure 3 and Table 4).

**Table 3:** Diagnostic panel in the discovery cohort

*m/z*	β	OR	95% CI	*P*-value
1895.3	0.104	1.110	(1.007-1.223)	0.036
2020.9	0.180	1.197	(1.045-1.371)	0.010
2080.7	-0.118	0.888	(0.797-0.990)	0.032
2656.8	-0.525	0.592	(0.467-0.750)	<0.001
3238.5	0.481	1.617	(1.330-1.966)	<0.001
Constant	-1.237	0.290		0.088

*P*-value lower than 0.05 was considered statistically significant. β, regression coefficient; OR, odds ratio; CI, confidence interval.

**Table 4:** Diagnostic performance of the diagnostic panel in the discovery and validation cohorts

	Diagnostic group	Real group		Accuracy rate	Sensitivity (95% CI)	Specificity (95% CI)	AUC (95% CI)	*P*-value
		CRC patients	Controls					
Discovery cohort	CRC patients	96	5	95.5%	96.0%	95.0%	0.982	<0.001
(n=200)	Controls	4	95		(92.2%-99.8%)	(90.7%-99.3%)	(0.965-0.998)	
Validation cohort	CRC patients	87	11	91.8%	95.6%	87.9%	0.932	<0.001
(n=182)	Controls	4	80		(91.6%-99.6%)	(81.5%-94.3%)	(0.892-0.973)	

### External validation of the peptide diagnostic panel

The same parameters generated from the discovery cohort were then used in the validation cohort to validate the diagnostic performance of the peptide diagnostic panel. The diagnostic panel yielded an accuracy of 91.8%, sensitivity of 95.6%, specificity of 87.9%, and AUC of 0.932 in the validation cohort, with similar results found in the discovery cohort (Figure 3 and Table 4).

### Peptide identification

By LC-MS/MS detection, the amino acid sequences of 3 peptides (peaks at *m/z* 1895.3, 2020.9, 3238.5) were identified as the partial sequences of complement component 4 (C4), complement component 3 (C3) and fibrinogen α chain (FGA), respectively (Table 5). However, we failed to identify the amino acid sequences of peptide peaks at *m/z* 2080.7 and 2656.8. The unknown modification on peptides may contribute to this phenomenon [34].

**Table 5:** Identified candidate peptide biomarkers for CRC

*m/z*	Sequence	Protein name
1895.3	R.NGFKSHALQLNNRQIR.G	Complement component 4 (C4)
2020.9	R.SSKITHRIHWESASLLR.S	Complement component 3 (C3)
2080.7	N/A	N/A
2656.8	N/A	N/A
3238.5	K.SYKMADEAGSEADHEGTHSTKRGHAKSRPV.R	Fibrinogen α chain (FGA)

## DISCUSSION

Various studies have suggested that serum peptidome is a promising tool for the effective identification of CRC patients [20–30]. Although the discriminatory peptides were not consistent among those studies because of the diverse methodology of sample preparation, measurement and/or data processing, there were some common features of those studies [35]. For example, all the diagnostic models yielded high accuracies. Moreover, fragment of C3 was reported as a candidate biomarker in three reports [25, 27, 30]. In our study, we utilized MB-WCX coupled with MALDI-TOF MS to analyse 382 serum samples. We established a promising diagnostic panel to diagnose CRC with 5 serum peptides (peaks at *m/z* 1895.3, 2020.9, 2080.7, 2656.8 and 3238.5). The diagnostic panel was able to discriminate CRC patients from healthy controls with high accuracy (discovery cohort: accuracy 95.5%, sensitivity 96.0%, specificity 95.0% and AUC 0.982; independent validation cohort: accuracy 91.8%, sensitivity 95.6%, specificity 87.9% and AUC 0.932). In addition, three diagnostic peptides were identified as fragments of C4, C3 and FGA, respectively. The identification and functional analysis of the discriminating peptides might provide new insights into cancer behaviours.

External validation is a critical step in introducing a new diagnostic model, as it evaluates the performance and transportability of a model using data that were not included in the model construction. Most of diagnostic models of CRC either had no validations [26], or had only internal validations, while only three studies were externally validated [23, 24, 30] (Table 6). In this study, we developed and validated the diagnostic panel with high sensitivity and specificity in both discovery and validation cohorts with relative large samples, suggesting that the established diagnostic panel may have a potential of high performance in generalization.

**Table 6:** The comparison of the present study with similar studies

Studies	MS methods	Population	Discovery cohort (CRC/control)	Validation cohort (CRC/control)	Validation methods	Sensitivity	Specificity	Identification
The present study	MALDI-TOF	Asians	100/100	91/91	External validation	95.6%	87.9%	C4, C3, FGA
Fan *et al* 2012 [20]	MALDI-TOF	Asians	38/32	34/33	Split samples	94.1%	100%	----
Deng *et al* 2014 [21]	MALDI-TOF	Asians	33/32	34/33	Split samples	100%	100%	----
De Noo *et al* 2006[22]	MALDI-TOF	Caucasians	66/50	----	Cross-validation	95.2%	90.0%	----
Engwegen *et al* 2006 [23]	SELDI-TOF	Caucasians	40/49	37/31	External validation	89.5%	88.9%	Apolipoprotein C1, A1
Liu *et al* 2009 [24]	SELDI-TOF	Asians	74/48	60/39	External validation	95.0%	94.78%	----
Pietrowska *et al* 2011[25]	MALDI-TOF	Caucasians	35/45	----	Cross-validation	68.6%	81.9%	C4A, C3
Zhai et al 2012 [26]	SELDI-TOF	Asians	73/26	----	No validation	----	----	STK4
Ward *et al* 2006 [27]	SELDI-TOF	Caucasians	62/31	----	Cross-validation	94.0%	96.0%	C3a, Apolipoprotein C1
Yu *et al* 2004[28]	SELDI-TOF	Asians	55/92	----	Cross-validation	89.0%	92.0%	----
Chen *et al* 2004 [29]	SELDI-TOF	Asians	55/92	----	Cross-validation	91.0%	93.0%	----
Habermann *et al* 2006 [30]	SELDI-TOF	Caucasians	58/32	38/21	External validation	96.8%	96.2%	C3a

SLEDI-TOF MS, surface-enhanced laser desorption/ionization time-of-flight mass spectrometry; MALDI-TOF MS, matrix-assisted laser desorption/ionization time-of flight mass spectrometry; STK4, serine/threonine kinase 4; C3, complement component 3; C4, complement component 4; FGA, Fibrinogen α chain.

Several potential peptide biomarkers, arising from apolipoprotein A-1, apolipoprotein C-1, C3, C4 and serine/threonine kinase 4 (STK4) have been identified by previous studies [23, 25–27]. Consistent with the reports of Pietrowska *et al* (2011) [25] and Ward *et al* (2006) [27], we also identified C3 peptides and C4 peptides as candidate biomarkers for CRC. C3 and C4 play critical roles in the activation of the complement system [36]. The excessive complement activation and complement deficiencies may contribute to several diseases and pathological conditions [37]. Increased complement activity was observed in cancer [38], while decreased complement activity has been observed in bacterial infections [39, 40]. Patients with active lupus erythematosus may have lower levels of C3 and C4 than healthy controls [41]. The complement system is a central part of immune system regarded as the first defence against “non-self” cells [42]. It contributes to immune cell activation, pathogen elimination and immune surveillance against cancer [43]. Neoplastic transformation of tumour cells can generate tumour-associated antigens that distinguish malignant cells form normal ones. The components of the complement play a role in anti-tumour immune response through complement-dependent cellular cytotoxicity (CDCC) [44]. Various studies demonstrated that certain tumour cells activate complements. Elevated levels of C3 are present in patients with ovarian cancer [45]. The lectin pathway of complement activation has been found to be significantly increased in patients with CRC [46]. High expression of complement regulatory proteins were associated with poor prognosis of CRC [47]. Our results indicated the possibility of use of complement-related proteins/peptides as new cancer biomarkers. A more systematic analysis of abnormalities in the levels of complement-related proteins/peptides occurring in serum of cancer patients is needed, which can also contribute to better understanding of the dynamic interplay between CRC and complements.

The peak at *m/z* 3238.5 identified as a fragment of FGA, showed a higher intensity in the CRC patients compared to the healthy controls. This peptide may reflect the status of high fibrinogen level in CRC patients. Fibrinogen is a serum protein secreted by hepatocytes and plays a central role in coagulation [48]. Elevated fibrinogen level is associated with malignant growth and hematogenous metastasis [49, 50]. The fibrinogen receptors of malignant cells can bind the fibrinogen. The excessive fibrinogen may act as a physical barrier that can protect the malignant cells from the NK-mediated killing. Thus, high fibrinogen level enhances the early survival of tumour cells by protecting malignant cells from eliminating by the innate immune system [51]. Several studies have reported that increase of plasma fibrinogen level in patients with various types of malignancy, including colorectal cancer [52–54], lung cancer [55], pancreas cancer [56], ovarian cancer [57] and gastric cancer [58].

It is suggested that higher or lower intensities of various peptides in cancer serum can be originated not only from up- or down-regulation of the parent proteins, but from cancer-specific exoprotease activities as well. It has been proved that profiling of exoproteases is altered in several cancers, e.g., ectopeptidasea CD10, CD13, CD26 and CD143 are up-regulated in gastric cancer [59] and methionine aminopeptidase 2 are up-regulated in colorectal cancer [60]. Our work was based on the hypothesis that the different peptide patterns observed in controls and cancer patients were caused by the cancer-specific exoprotease activities. Although C3, C4 and FGA are not CRC-specific biomarkers, the profiling of CRC-specific peptide pattern and/or CRC-specific peptide sequence has potential value in diagnosis of CRC. Additionally, biomarker discovery at OMICs level is moving away from the idealized single cancer-specific biomarker. Seldom there is a single biomarker with both high levels of specificity and sensitivity that can meet the requirement for routine clinical practices, due to the molecular heterogeneity of tumours [19]. Although an individual biomarker maybe specific and sensitive only for a certain molecular aetiology, combinations of many markers can transcend the heterogeneity to reach higher specificity and sensitivity. Thus, the cancer-specific peptide panel may play a crucial role in the diagnosis of cancers.

There were several limitations of this study must be demonstrated. Due to the small sample size of the early-stage patients in the cohorts, we were unable to compare the differences in peptides between the early-stage patients and the healthy controls. This weakness may restrict the clinical value of the panel in early detection of CRC. Further case-control study including more early-stage patients or a prospective cohort study in design should be performed to determine the diagnostic value of the peptide panel. In addition, the peptides are fragments of proteins involved in acute phase and inflammatory response. Thus, the specificity of proposed biomarkers may be doubted. Further studies including functional analysis are essential to answer this question. Finally, because of the identification of proteases was not our primary goal of the study, we did not research the cancer-specific proteases activities. Studies that focus on cancer-specific proteases activities may shed light on CRC pathophysiology and find possible targets for CRC therapy.

In conclusion, using MALDI-TOF MS and LC-MS/MS, we have characterized 5 peptides (peaks at *m/z* 1895.3, 2020.9, 2080.7, 2656.8 and 3238.5) to be novel candidate biomarkers for CRC diagnosis. We have constructed a peptide diagnostic panel that could diagnose CRC with an accuracy of 91.8%, sensitivity of 95.6%, specificity of 87.9% and AUC value of 0.932. As a diagnostic panel, there is potential for this method to provide an *in vitro* diagnosis tool for CRC. We have also identified the amino acid sequences of peptides fragments (peaks at *m/z* 1895.3, 2020.9 and 3238.5) from C3, C4 and FGA, suggesting that increased levels of C3, C4 and FGA might associate with the pathogenesis of CRC.

## MATERIALS AND METHODS

### Participants

The discovery cohort containing 100 patients with CRC and 100 healthy individuals recruited from Beijing *Shijitan* Hospital between January 2013 and December 2014. The independent validation cohort consisted of 91 patients with CRC and 91 healthy participants recruited from another hospital, Beijing Friendship Hospital, between March 2011 and December 2012. Each CRC patient underwent a colonoscopic biopsy and was diagnosed by two senior pathologists according to World Health Organization (WHO) diagnostic criteria [61]. Pathological samples were classified according to the TNM stage classification system [61]. All healthy controls and CRC patients were gender- and age- matched.

Controls should meet the following inclusion criteria: (1) older than 18 years old, (2) capable of giving informed consent, and (3) confirmed to be healthy without any diseases detected during physical examination. Controls were excluded if they met any of the following criteria: (1) had previous history of cancer, (2) had used any drugs, and (3) were pregnant or breastfeeding.

The patients with CRC met the following inclusion criteria: (1) older than 18 years, (2) capable of giving informed consent, and (3) had colorectal cancer. Patients were excluded if they had any of the following: (1) other cancers; (2) a history of other cancers; (3) a history of radiotherapy or chemotherapy; (4) any severe diseases concerning the cardiovascular system, respiratory system, genitourinary system, digestive system or circulatory system; or (5) a systemic infection. The study was approved by the ethical committee of Capital Medical University, Beijing.

### Collection of serum samples

Fasting blood samples from the participants were collected in the morning and allowed to clot at 37°C for 30 mins. All blood samples from the CRC patients were obtained before the colorectal surgery. Serum was then separated by centrifugation at 3000 rpm for 15 mins and then stored at -80°C until further analysis.

### Peptides fractionation

All serum samples were fractionated using MB-WCX kit, according to the instructions provided by the supplier (ClinProt^TM^, Bruker Daltonics, Billerica, MA, USA) [62]. The samples were purified and isolated in three steps: binding, washing, and elution. Firstly, 10 μl beads, 10 μl MB-WCX binding solution and 5 μl serum were added in a 0.2 ml polypropylene tube, mixed by pipetting up and down several times, and then incubated for 5 min. Secondly, the tubes were placed on a magnetic bead separator for 1 min and the beads were grasped on the tube wall. The supernatant was removed and 100 μl of magnetic bead washing solution was added, and mixed thoroughly. After three times washing, the bound peptides were eluted from the magnetic beads by 5 μl of eluting solution.

### Peptides profiling by MALDI-TOF MS and processing of spectral data

A portion of the eluted sample was diluted (1:10) in α-cyano-4-hydroxycinnamic acid (CHCA) matrix solution (0.5 g/L CHCA in acetonitrile/water 1:1 *v/v* containing 0.1% trifluoroacetic acid) (Sigma-Aldrich, St, Louis, MO, USA). Then 1 μl of the mixture was spotted onto a MALDI-TOF MS target (Bioyong Tech, Beijing, China) and dried at room temperature before analysis. Spectral profiles were acquired using a MALDI-TOF MS (Clin-TOF^TM^, Bioyong Tech, Beijing, China). The instrument was calibrated using a mix of commercial peptide and protein calibration standards (Sigma-Aldrich, St, Louis, MO, USA) prepared in the same matrix solution as above. Spectra were acquired automatically in a 1,000-10,000 mass-to-change ratio (*m/z*) range in linear mode. Each spectrum was the sum of 1,000 laser shots per spotted sample, delivered to different locations on the spot in 10 sets of 100 shots (at a laser frequency of 10 Hz).

All spectra obtained from the MALDI-TOF MS were pre-processed using BioExplorer^TM^ 2.0 (Bioyong Tech, Beijing, China) [63]. In brief, the background was estimated and then subtracted from each spectrum based on local noise estimators. Peaks were detected using a signal-to-noise ratio (S/N) cut-off of 5.0, which was found to be a good compromise between overdetection and sensitivity. To align the spectra, a mass shift of no more than 0.1% was determined. Smoothing was applied by averaging the intensities within a 5-point width moving window followed by baseline subtraction using an algorithm based on finding the lowest points between dominant local intensity maxima within a particular mass window. Normalization was performed by dividing the intensity of each data point in a spectrum by the sum of all intensities in that spectrum.

### Peptide identification by LC-MS/MS

The amino acid sequences of the candidate peptides were identified using a nano-liquid chromatography-electrospray ionization-tandem mass spectrometry (nano-LC/ESI-MS/MS) consisting of an Aquity^TM^ UPLC system (Waters, Milford, MA, USA) and a LTQ Orbitrap XL mass spectrometer (Thermo Fisher Scientific, Pittsburgh, PA, USA) equipped with a nano-ESI source. A sample of 5 μl solution was injected on the column. The mobile phase A, mobile phase B, flow rate and gradient elution were operated according to the published methods [16]. The obtained samples were further analysed using the MS/MS instrument. The Mascot 2.4.1 (Matrix Science, London, UK) was used to search the database. The results were restricted to “*Homo sapiens*” with the peptide mass tolerance set to ±20 ppm and the fragment mass tolerance set to ± 0.2 Da.

### Statistical analysis

Statistical analyses and displays were performed using SPSS 19.0 (IBM Corporation, New York, USA) and GraphPad Prism 5.0 (GraphPad, San Diego, USA). Normality of variables was tested by Shapiro-Wilk test. Student's *t* test was used to compare normally distributed continuous data, and the Mann-Whitney U-test was used for non-normally distributed continuous data. The chi-square test was used for the analysis of categorical data. Differential peptide peaks were used to establish a diagnostic panel by multivariate binary logistic regression. Receiver operating characteristic (ROC) curves and the area under the curve (AUC) were used to assess the diagnostic performance of the model. *P* < 0.05 was considered statistically significant (two-tailed).

## Abbreviations

CRC: colorectal cancer
MB-WCX: weak cation exchange magnetic beads
MALDI-TOF MS: matrix-assisted laser desorption/ionization time-of-flight mass spectrometry
LC-MS/MS: liquid chromatography-tandem mass spectrometry
C3: complement component 3
C4: complement component 4
FGA: fibrinogen α chain
FOBT: fecal occult blood test
CEA: carcinoembryonic antigen
